# Influence of α-terpineol on the growth and morphogenesis of *Penicillium digitatum*

**DOI:** 10.1186/s40529-015-0116-4

**Published:** 2015-12-13

**Authors:** Guo-xing Jing, Neng-guo Tao, Lei Jia, Hai-en Zhou

**Affiliations:** School of Chemical Engineering, Xiangtan University, Xiangtan, 411105 People’s Republic of China

**Keywords:** Antifungal activity, Cell membrane integrity, *P. digitatum*, α-Terpineol

## Abstract

**Background:**

Plant essential oils could act effectively against postharvest diseases, α-terpineol, a typical terpenoid of plant essential oils, exhibited strong antifungal activity in against *Penicillium italicum*, but the possible action mechanism remains undetermined. In present study, α-terpineol was evaluated for antibacterial activity against *Penicillium digitatum* along with the mode of their antibacterial action.

**Results:**

The results showed that mycelial growth of *P. digitatum* was strongly inhibited by α-terpineol, with the minimum inhibitory concentration (MIC) and minimum fungicidal concentration (MFC) of 2.00 and 8.00 µl/ml, respectively. Scanning electron microscopy observation revealed that α-terpineol obviously altered the morphology of *P. digitatum* hyphae by causing the loss of cytoplasm and distortion of mycelia. A rapid increase in the membrane permeability of *P. digitatum* was observed after treated with MIC or MFC of α-terpineol, evidenced by the release of cell constituents, the extracellular conductivity, and the extracellular pH. In addition, α-terpineol apparently induced a decrease in total lipid contents of *P. digitatum* cells, indicating the destruction of cell membrane structures after treatment.

**Conclusions:**

Based on our study, α-terpineol might affect the cell wall synthesis and lead to the disruption of cell wall. The cell wall disruption affected fungal morphogenesis, the integrity of membrane and leakage of intracellular components, these results suggested that α-terpineol treatment inhibited the growth of *P. digitatum*.

## Background

Citrus fruit can be infected by many fungal pathogens in the postharvest storage. Among them, *Penicillium digitatum* is the most devastating pathogen and causing about 90 % of production losses during postharvest handling of the fruit (Droby et al. 2008; Liu et al. 2010). Currently, this disease is primarily controlled by applying synthetic fungicides (Cañamás et al. 2008). However, considerable scientific interest has been directed toward alternative substances, which were capable of controlling this disease by decreasing the resistance of postharvest fungal pathogens, and the fungicides were primary concerned for human safety and environmental protection in recent reports (Sharma and Tripathi 2008; Viuda-Martos et al. 2008).

The use of plant essential oils as an alternative to synthetic fungicides has attracted keen interest in the past decades (du Plooy et al. 2009; Askarne et al. 2012). α-terpineol, a type of terpenoids constituting in many plant essential oils, reportedly exhibits strong antifungal activity (Park et al. 2009). Scora and Scora (1998) found that the inhibition zones of *P. digitatum*, *P. italicum* and *P. ulaiense* upon the addition of α-terpineol at a concentration of 5 μl per plate were 87, 71, and 106 mm^2^, respectively. In another report, α-terpineol at a concentration of 400 μg/ml reduced by 46 % the radial growth of *P. digitatum* (Daferera et al. 2000). However, the mechanism which α-terpineol inhibited the growth of *P. digitatum* remains undetermined. Therefore, this study aims to investigate the antifungal activity of α-terpineol against the mycelial growth of *P. digitatum*, and to elucidate its possible mode of action.

## Methods

### Pathogens

The fungal pathogen *P. digitatum* was isolated from infected citrus fruit and maintained on potato dextrose agar (PDA) at 25 ± 2 °C. The spores’ concentration was adjusted to 5 × 10^5^ cfu/ml using a hamacytometer.

### Chemicals

α-Terpineol (90 %) was obtained from Sigma-Aldrich (St. Louis, MO, USA). Cholesterol (95 %) and phosphovanillin (98 %) were purchased from TCI Shanghai (Shanghai, China).

### Measurement of mycelial growth

Effects of α-terpineol on the mycelial growth of *P. digitatum* were evaluated by the poisoned food technique (Sharma and Tripathi 2008). PDA (20 ml) was poured into sterilized Petri dishes (90 mm diameter) and measured amount of α-terpineol was added to give desired concentrations (0.00, 0.25, 0.50, 1.00, 2.00, 4.00 and 8.00 μl/ml). In media, 0.05 % (v/v) Tween-80 was added. Then the colony diameter was measured after 2 days incubation at 25 ± 2 °C. Each treatment was performed in triplicates. The percentage of inhibition of mycelial growth (MGI) was calculated according to the following formula (Yahyazadeh et al. 2008):

$${\text{MGI (}}\% ) = [({\text{dc}} - {\text{dt}})/{\text{dc}}] \times 100$$

where dc (cm) is the mean colony diameter for the control sets and dt (cm) is the mean colony diameter for the treatment sets. The lowest concentration that completely inhibits the growth of the fungus after 2 days of incubation is considered the minimum inhibitory concentration (MIC). Minimum fungicidal concentration (MFC) is regarded as the lowest concentration that prevents growth of the pathogen after a following 72 h incubation at 25 ± 2 °C.

### Scanning electron microscopy (SEM) observation

The 4-day-old fungal cultures on PDA treated with α-terpineol at various concentrations (0, MIC and MFC) were used for all SEM observations (Helal et al. 2007; Yahyazadeh et al. 2008). About 5 × 10 mm segments were cut from cultures growing on PDA plates and promptly placed in vials containing 3 % (v/v) glutaraldehyde in 0.05 M phosphate buffer (pH 6.8) at 4 °C. Samples were kept in this solution for 48 h for fixation and then washed with distilled water three times for 20 min each. Following which they were dehydrated in an ethanol series (30, 50, 70, and 95 %, v/v), for 20 min in each alcohol dilution and finally with absolute ethanol for 45 min. Samples were then critical point dried in liquid carbon dioxide. Fungal segments were placed in desiccators until further use. Following drying, samples prepared were mounted on standard 1/2 in SEM stubs using double-stick adhesive tabs and coated with gold–palladium electroplating (60 s, 1.8 mA, 2.4 kV) in a Polaron SEM Coating System sputter coater. All samples were viewed in a JEOL JSM-6360LV SEM (JEOL, Tokyo, Japan) operating at 25 kV at 5000× level of magnification.

### Determination of the release of cell constituents

The release of cell constituents was measured according to the method described previously (Paul et al. 2011) with minor modifications. Briefly, *P. digitatum* cells from 100 ml potato dextrose broth (PDB) were initially collected by centrifugation at 4000*g* for 20 min, washed three times with sterilized double distilled water, and resuspended in 100 ml phosphate buffered saline (pH 7.0). After that, the suspensions were treated with α-terpineol at various concentrations (0, MIC and MFC) for 0, 30, 60 and 120 min. Then, 1 ml of supernatant was used to measure the absorbance at 260 nm with a UV-2450 UV/Vis spectrophotometer [SHIMADZU international trade (Shanghai) Co. Ltd, Shanghai, China].

### Measurement of extracellular conductivity and extracellular pH

The measurement of extracellular conductivity and extracellular pH of *P. digitatum* cells was carried out using a DDS-12DW conductivity meter (Bante Instrument Co., Ltd., Shanghai, China) and a Delta-320 pH-meter (Mettler-Toledo, Greifensee, Switzerland) following the instructions, respectively. The *P. digitatum* cells treated by MIC or MFC of α-terpineol were selected at 0, 30, 60 and 120 min of treatment. Control flasks without α-terpineol were also tested.

### Determination of total lipid content

Total lipid content of *P. digitatum* cells with α-terpineol at various concentrations (0, MIC and MFC) was determined using phosphovanillin method (Helal et al. 2007). The 2-day-old mycelia from 50 ml PDB was collected and centrifuged at 4000*g* for 10 min. Then the samples were dried with a vacuum freeze drier for 4 h. About 0.1 g of dry mycelia were homogenized with liquid nitrogen and extracted with 4.0 ml of methanol–chloroform–water mixture (2:1:0.8, v/v/v) in a clean dry test tube with vigorous shaking for 30 min. The tubes were centrifuged at 4000*g* for 10 min. The lower phase containing lipids was thoroughly mixed with 0.2 ml saline solution and centrifuged at 4000*g* for 10 min. Then, an aliquot of 0.2 ml chloroform and lipid mixture was transferred to a novel tube and 0.5 ml H_2_SO_4_ was added, heated for 10 min in a boiling water bath. After that, 3 ml phosphovanillin was added and shake vigorously, and then incubated at room temperature for 10 min. The absorbance at 520 nm was utilized to calculate total lipid content from the standard calibration curve using cholesterol as a standard.

### Statistical analysis

All experiments were performed as three biological replicates and statistical analysis (ANOVA) was performed using Statistical Package for the Social Science (SPSS) statistical software package release 16.0 (SPSS Inc., Chicago, IL, USA). Statistical significance was set at *P* < 0.05 and *P* < 0.01.

## Results and discussion

### Inhibition of mycelial growth

As shown in Table 1, the mycelial growth of *P. digitatum* was affected by α-terpineol in a dose-dependent manner (*P* < 0.01). High α-terpineol concentration (≥2.00 μl/ml) completely inhibited the mycelial growth of *P. digitatum*, whereas α-terpineol at a concentration lower than 1.00 μl/ml only showed moderate antifungal activity against *P. digitatum* after 2 days of incubation. After 4 days of incubation, the mycelial growth of *P. digitatum* was still totally inhibited by the application of 8.00 μl/ml α-terpineol. Thus, the MIC and MFC of α-terpineol for *P. digitatum* were measured to be 2.00 and 8.00 μl/ml, respectively.

**Table 1:** Effect of α-terpineol on the mycelial growth of *P. digitatum*

Concentration (μl/ml)	Percentage of inhibition of mycelial growth (%)
0.00	0.00 ± 0.00^a,A^
0.25	8.33 ± 0.00^b,B^
0.50	11.11 ± 4.81^c,B,C^
1.00	22.22 ± 9.62^c,C^
2.00	100.00 ± 0.00^d,D^
4.00	100.00 ± 0.00^d,D^
8.00	100.00 ± 0.00^d,D^

Each value is presented as mean ± standard deviation (n = 3). Different letters of a, b, c and d are significantly different according to Duncan’s multiple range test at *P* < 0.05, A, B, C and D are highly significant difference between the control and α-terpineol treatment at *P* < 0.01

This study proved the effectiveness of α-terpineol on inhibiting the mycelial growth of *P. digitatum*, and the inhibitory effect was positively correlated with the α-terpineol concentration. The MIC of only showed partially antifungal activity against *P. digitatum* after 4 days of incubation, but the MFC of α-terpineol totally inhibited the growth of *P. digitatum* (Table 1). These results were consistent with those of previous studies describing the antifungal activity of α-terpineol (Scora and Scora 1998; Daferera et al. 2000; Park et al. 2009), α-terpineol treatment could make a major contribution to limiting the spread of the pathogen by lowering the spore load, and the presence of aromatic ring with a phenolic hydroxylic group in α-terpineol were suggested to be responsible for the fungitoxicity of the essential oils (Daferera et al. 2000).

### Change in mycelial morphology, cell constituents, extracellular conductivity and extracellular pH

**Fig. 1:**
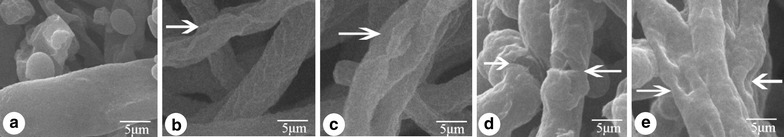
SEM image of *P. digitatum*. **a** Mycelia of untreated (control) *P. digitatum* with linearly shaped hyphae; **b**, **c** *P. digitatum* treated with MIC of α-terpineol (*arrows* refer to the morphologic changes in the hyphae, such as warty surfaces); **d**, **e** *P. digitatum* treated with MFC of α-terpineol (*arrows* refer to the morphologic changes of collapsed cell and irregular branching)

The effect of α-terpineol on the morphology of *P. digitatum* was examined using SEM (Fig. 1). The control fungus grown on potato dextrose agar (PDA) had normal, tubular, regular, and homogenous hyphae (Fig. 1a). All mycelia of *P. digitatum* treated with MIC or MFC of α-terpineol showed considerable changes in hyphal morphology. *P. digitatum* treated with MIC of α-terpineol showed the loss of linearity and a warty surface of mycelia (Fig. 1b, c). Moreover, the hyphae of *P. digitatum* treated with MFC of α-terpineol exhibited abnormal branching (Fig. 1d), and shrunken and distorted mycelia (Fig. 1e).

**Fig. 2:**
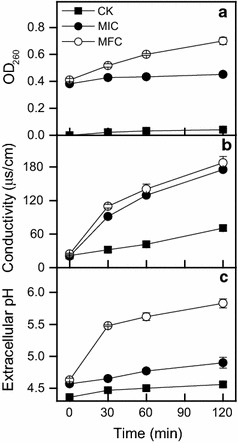
Effects of α-terpineol on the cell constituents’ release (**a**), extracellular conductivity (**b**), and extracellular pH of *P. digitatum* cells (**c**). *Bars* represent standard deviations of the means

The release of cell constituents significantly increased (*P* < 0.05) when *P. digitatum* was treated with early exposure of MIC or MFC (Fig. 2a). The OD_260_ value in *P. digitatum* suspensions with MIC of α-terpineol for 30 min was 0.43, which was significantly higher (*P* < 0.05) than that of the control (0.02) but significantly lower (*P* < 0.05) than that with MFC of α-terpineol (0.52). The OD_260_ value of *P. digitatum* suspensions treated with MIC of α-terpineol maintained a smooth ascending trend after 30 min of exposure, whereas that with MFC continuously increased after 30 min of exposure, and reached to the highest absorbence of 0.70 at 120 min of exposure.

Although α-terpineol treatment did not affected the extracellular conductivity at initial exposure time, α-terpineol caused a significant increase after 30 min incubation (Fig. 2b). Moreover, the extracellular conductivity in *P. digitatum* suspensions with MIC or MFC of α-terpineol remained a comparable level during the entire period. After 120 min of exposure, the extracellular conductivity in *P. digitatum* suspensions with MFC and MIC of α-terpineol were 187.7 and 175.5 μs/cm, respectively, which were significantly higher (*P* < 0.05) than that in control (70.8 μs/cm).

The extracellular pH in the control maintained a stable level during the entire incubation. The extracellular pH in *P. digitatum* suspensions treated by α-terpineol is presented in Fig. 2c. Conversely, the extracellular pH of *P. digitatum* suspensions with MIC or MFC of α-terpineol rapidly increased in the first 30 min of exposure, and followed by a moderate ascending trend in the subsequent stage. The extracellular pH values in *P. digitatum* suspensions after incubation with MFC and MIC of α-terpineol for 120 min were 5.83 and 4.90, respectively, which were significantly higher than that of the control (4.56) (*P* < 0.05).

The mechanism by which α-terpineol inhibited the growth of *P. digitatum* was poorly understood. It is generally accepted that the lipophilicity of terpenoids enabled them to preferentially partition from an aqueous phase into membrane structures of the fungi, resulted in membrane expansion, increased membrane fluidity and permeability, and induced the leakage of ions and other cellular contents (Burt 2004; Fadli et al. 2012; Bajpai et al. 2013). In the present study, the rough surface and shrinkage of cells were observed in the hyphae treated with α-terpineol, this SEM result indicated that the mode of fungitoxicity action of α-terpineol against *P. digitatum* might also be through cell growth hindrance. The loss of linear shape (Fig. 1b, c) and the branching of terminal hyphae (Fig. 1d, e) were showed that α-terpineol treatment might affect the apex growth of terminal hyphae, alter the normal assembly of the wall components and lost the directionality of vesicles. The application of α-terpineol could result in releasing parietal materials at positions where exocytosis did not normally taken place, the changes in the wall surface might inhibit the enzymatic reactions of cell wall synthesis (Romagnoli et al. 2005). These observations indicated that the mode of antifungal activity of α-terpineol was a result of attack of oil on the cell wall, retraction of cytoplasm in the hyphae and ultimately death of the mycelium (Ghfir et al. 1997).

Membrane permeability parameters, including loss of 260 nm absorbing materials, change in extracellular conductivity, and extracellular pH, were used to reflect the integrity of membrane. These parameters are commonly used to indicate gross and irreversible damage to the cytoplasmic and plasma membranes (Turgis et al. 2009; Paul et al. 2011; Shao et al. 2013). The addition of α-terpineol caused the rapid loss of the absorbing material at 260 nm in the tested fungal suspensions (Fig. 2a). The maximum release of cell constituents was observed in the *P. digitatum* cell suspensions treated with MFC of α-terpineol, showing an absorbance of 0.70 after 120 min of exposure. The extracellular conductivity in the *P. digitatum* suspensions with α-terpineol was higher than that of the control at all times, the values increased significantly after 30 min of exposure and positively correlated with the α-terpineol concentration (Fig. 2b), indicating rapid leakage of metal ions tested after the membrane disruption. In addition, remarkable increase of extracellular pH in *P. digitatum* suspensions indicating the occurrences of an irreversible impairment of pH homeostasis after α-terpineol treatment (Fig. 2c).

These findings might be attributed to the essential oil’s lipophilic properties, the oil treatment made α-terpineol permeable to the cell wall and assisted in the accumulation of polysaccharides under water stress conditions (Sharma and Tripathi 2008). The cytoplasmic contents of phospholipids, fatty acids, proteins, and polysaccharides might be leaked out after the disruption of membrane fluidity and integrity, this result might be responsible for the establishment of antifungal activity (Marquis et al. 2003; Oonmetta-aree et al. 2006). Such modifications induced by α-terpineol might be related to the interference of α-terpineol with enzymatic reactions of wall synthesis, which affected fungal morphogenesis and growth.

### Total lipid content

**Fig. 3:**
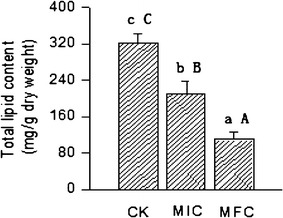
Effect of α-terpineol on the total lipid content of *P. digitatum*. *Bars* represent standard deviations of the means. *Different letters* of *a*, *b* and *c* are significantly different according to Duncan’s multiple range test at *P* < 0.05, *A*, *B* and *C* are highly significant difference between the control and α-terpineol treatment at *P* < 0.01

The total lipid of *P. digitatum* cells was significantly decreased during incubation after α-terpineol treatment (Fig. 3). After 120 min incubation, the total lipid contents of *P. digitatum* cells treated with MFC and MIC of α-terpineol were 109.87 and 210.41 mg/g dry weight, respectively, which are significantly lower (*P* < 0.01) than that of the control (323.31 mg/g dry weight).

Lipids were the main components of biological membranes (Watkins et al. 2011). The decrease in lipid content suggested that the membrane stability was reduced and the permeability to water-soluble materials was increased (Helal et al. 2007). In our study, the application of α-terpineol significantly decreased the lipid contents of *P. digitatum* (Fig. 3), and we hypothesized that α-terpineol could interfere with the phospholipid bilayers of membranes (Xing et al. 2014) and decrease the lipids content of *P. digitatum*. Thus, this result confirmed that α-terpineol could damage the cell membrane integrity of *P. digitatum*, affected the cell membrane structure after the disruption of cell wall and inhibited the growth of *P. digitatum*.

## Conclusion

Based on the present study, it could be concluded that α-terpineol possess fungitoxic activities inhibiting the growth of *P. digitatum*, leading to the disruption of cell wall, and subsequently resulting in irreversible deleterious morphological alterations, disturbance of membrane structure, increase in membrane fluidity, leakage of ions and other cell contents. The present data indicated that α-terpineol could become a possible alternative to synthetic fungicides in the fight against *P. digitatum*.
